# Maternal immune activation-induced proBDNF-mediated neural information processing dysfunction at hippocampal CA3-CA1 synapses associated with memory deficits in offspring

**DOI:** 10.3389/fcell.2022.1018586

**Published:** 2022-11-11

**Authors:** Wei Sun, Yazi Mei, Xiaoliang Li, Yang Yang, Lei An

**Affiliations:** ^1^ Department of Pediatric, The First Affiliated Hospital, Guizhou University of Traditional Chinese Medicine, Guiyang, Guizhou, China; ^2^ Behavioral Neuroscience Laboratory, The First Affiliated Hospital, Guizhou University of Traditional Chinese Medicine, Guiyang, Guizhou, China; ^3^ Graduate School, Guangzhou University of Chinese Medicine, Guangzhou, China; ^4^ Department of Neurology, Jinan Geriatric/Rehabilitation Hospital, Jinan, China; ^5^ Department of Neurology, The First Affiliated Hospital, Guizhou University of Traditional Chinese Medicine, Guiyang, Guizhou, China

**Keywords:** hippocampus, maternal immune activation, neural information flow, ProBDNF, schizophrenia

## Abstract

Prenatal exposure to maternal infection increases the risk of offspring developing schizophrenia in adulthood. Current theories suggest that the consequences of MIA on mBDNF secretion may underlie the increased risk of cognitive disorder. There is little evidence for whether the expression of its precursor, proBDNF, is changed and how proBDNF-mediated signaling may involve in learning and memory. In this study, proBDNF levels were detected in the hippocampal CA1 and CA3 regions of male adult rats following MIA by prenatal polyI:C exposure. Behaviorally, learning and memory were assessed in contextual fear conditioning tasks. Local field potentials were recorded in the hippocampal CA3-CA1 pathway. The General Partial Directed Coherence approach was utilized to identify the directional alternation of neural information flow between CA3 and CA1 regions. EPSCs were recorded in CA1 pyramidal neurons to explore a possible mechanism involving the proBDNF-p75^NTR^ signaling pathway. Results showed that the expression of proBDNF in the polyI:C-treated offspring was abnormally enhanced in both CA3 and CA1 regions. Meanwhile, the mBDNF expression was reduced in both hippocampal regions. Intra-hippocampal CA1 but not CA3 injection with anti-proBDNF antibody and p75^NTR^ inhibitor TAT-Pep5 effectively mitigated the contextual memory deficits. Meanwhile, reductions in the phase synchronization between CA3 and CA1 and the coupling directional indexes from CA3 to CA1 were enhanced by the intra-CA1 infusions. Moreover, blocking proBDNF/p75^NTR^ signaling could reverse the declined amplitude of EPSCs in CA1 pyramidal neurons, indicating the changes in postsynaptic information processing in the polyI:C-treated offspring. Therefore, the changes in hippocampal proBDNF activity in prenatal polyI:C exposure represent a potential mechanism involved in NIF disruption leading to contextual memory impairments.

## Introduction

Epidemiological and experimental evidence suggests that adverse events occurring during the prenatal and early postnatal periods are associated with the development of psychiatric disorders (Brown and Derkits, 2010; Cannon et al., 2014). Infections during pregnancy are widely reported to increase the risk of a variety of psychiatric illnesses including autism, depression and schizophrenia (Jiang et al., 2016; Brown and Meyer, 2018; Gustafsson et al., 2018). Polyinosinic:polycytidylic acid (PolyI:C), a synthetic analog of double-stranded RNA (dsRNA) that targets and stimulates Toll-like receptor 3, is a potent stimulator of the immune system. PolyI:C-induced maternal immune activation (MIA) can initiate inflammatory responses and results in a variety of dose-dependent aberrant behavior responses in the offspring (Cunningham et al., 2007; Lipina et al., 2013). Given that polyI:C treatment leads to distinct behavioral and cognitive pathological symptoms depending on the precise prenatal timing, the animal polyI:C model provides a unique opportunity to link specific neuronal and neurochemical dysfunctions with different forms of psychosis-related behavior. This may readily set the stage for specific interventions targeting different neuronal and neurochemical systems, attempting to normalize behavioral and cognitive dysfunctions by symptomatic or preventive treatments (Meyer and Feldon, 2012).

A variety of evidence shows that brain-derived neurotrophic factor (BDNF) is crucial in the pathophysiology of mental diseases (Nagahara and Tuszynski, 2011; Cattaneo et al., 2016; Yang et al., 2017). Mature BDNF (mBDNF) is initially synthesized as the precursor protein, preproBDNF, which is processed to proBDNF. After cleavage of the signal peptide, proBDNF is converted into mBDNF by intracellular and extracellular proteases (Yang et al., 2017). A meta-analysis demonstrates that decreased peripheral mBDNF level is significantly linked to schizophrenia, thereby supporting the neurotrophins hypothesis of schizophrenia psychosis (Rodrigues-Amorim et al., 2018). Juvenile offspring of poly (I:C)-treated pregnant animals displayed cognitive deficits and a significant decrease in BDNF-TrkB (tyrosine kinase receptor) signaling in mouse prefrontal cortex (Han et al., 2016). Interestingly, oral treatment of the TrkB agonist 7,8-dihydroxyflavone during adolescence led to the prevention of behavioral abnormalities and enhanced BDNF-TrkB signaling and PV immunoreactivity of the MIA offspring. It clearly suggests that decreased BDNF-TrkB signaling is a key pathway involved in the cognitive deficits of MIA offspring (Han et al., 2016). A recent study found that compared with those from the control group, mBDNF levels in the parietal cortex of schizophrenia patients were significantly lower, whereas in the same region, proBDNF expression levels were abnormally higher. Differently, the cerebellar proBDNF levels of schizophrenia showed to be significantly reduced (Yang et al., 2017). ProBDNF exhibits the opposing effects to mBDNF on the functioning of neuronal development and synaptic plasticity (Yang et al., 2009; Yang et al., 2014), and these effects are most pronounced in the pathogenesis of neurodegenerative diseases (Buhusi et al., 2017; Wang et al., 2021). Moreover, proBDNF binds to the neurotrophin receptor p75^NTR^, thereby resulting in the activation of apoptosis pathway (Liu et al., 2018; Yang et al., 2021). Our recent studies also indicate that proBDNF is a neurotrophin that manifests its effects mainly during the early prenatal period (Sun et al., 2018a; Sun et al., 2020; Sun et al., 2021a). For example, the peak expression of proBDNF during perinatal period can facilitate the NMDAR-dependent short-lasting synaptic plasticity, which is accompanied by inhibiting neuronal migration and axonal retraction (Sun et al., 2021a). Therefore, the central role of proBDNF appears to be altered by maternal infection and may be associated with abnormal brain development. The dominance of proBDNF in both maternal and fetal parts of the placenta in the hyperhomocysteinemia-induced schizophrenia animal model suggests that the dynamic balance between BDNF isoforms produced during the processing plays an important role in neuronal plasticity and cognitive development (Arutjunyan et al., 2020). Furthermore, the interaction of proBDNF with the p75^NTR^ initiates apoptosis and causes epileptic seizure activity in the brain due to the up-regulated production of IL-1β (Volosin et al., 2008; Friedman, 2010; Patterson, 2015). The IL-1β activation of neuronal IL-1R1 signaling can modulate intracellular Ca^2+^ (Srinivasan et al., 2004), activation of postsynaptic receptors (Bertani et al., 2017) and BDNF-dependent synaptic signaling and function (Tong et al., 2012; Cai et al., 2014; Patterson, 2015). Thus, abnormal activation of these signaling pathways during neuronal development by proBDNF may impinge on multiple developmental mechanisms, resulting in the perturbation of neuronal differentiation, dendritic and axonal growth, myelination and synapse formation and undermining cognitive and behavioral performance (Sun et al., 2018a; Sun et al., 2021a; Sun et al., 2021b; Yang et al., 2021). However, little is known about how proBDNF is regulated by maternal infection and how this increases the risk for neurodevelopmental disorders.

Cerebral neurons have locally synchronous or oscillatory patterns of activity, and the temporal dynamics reflect different behavioral states (Salinas and Sejnowski, 2001; Schnitzler and Gross, 2005). Studies ranging from single-unit recordings in animals to electroencephalography studies in humans have demonstrated that correlated fluctuations are important for controlling the oscillation of information flow in the brain (Salinas and Sejnowski, 2001). Significantly, the specific cognitive impairments and psychotic features of schizophrenia indicate the fundamental deficits in processing information by neural circuits in the central nervous system (CNS) (Kocsis et al., 2013; McNally and McCarley, 2016; Van Derveer et al., 2021). Structural and functional abnormalities of the hippocampal circuits are the feature of major neuropsychiatric disorders with adult onsets and are considered to underlie the cognitive impairments associated with neurological diseases (Harrisberger et al., 2015; Ott et al., 2019). It is well known that hippocampal activity provides a powerful heterosynaptic learning rule for long-term gating of information flow at the CA3-CA1 synapses (Colgin et al., 2009; Jackson et al., 2014; Basu et al., 2016). Importantly, the functional relevance of hippocampal oscillations in animals with schizophrenia had been found to reproduce the stereotypic structural damage obtained from the hippocampal circuit of schizophrenic humans (Berretta et al., 2001; Keilhoff et al., 2004; Berretta and Benes, 2006). Furthermore, BDNF signaling is a key regulator of spatial cognition within these circuits. For example, by affecting the firing of GABAergic interneurons, BDNF plays an important role in regulating network oscillations in the hippocampus (Holm et al., 2009). Infusion of proBDNF is able to enhance neuronal correlate with fear extinction behavior without affecting basal firing rate in the infralimbic region of rodents (Sun et al., 2021b). Endogenous mBDNF signaling is necessary for strategy set-shifting (D’Amore et al., 2013), while both endogenous and exogenous proBDNF infusions into the dorsal striatum facilitate flexible behavior (Sun et al., 2020). However, recent research from our group found that both endogenous and intra-hippocampal proBDNF could elevate the spike frequency of pyramidal neurons, resulting in an improvement of contextual fear memory formation (Sun et al., 2019; Sun et al., 2021a). Therefore, it is needed to examine the hypothesis of whether maternal infection ultimately alters proBDNF-mediated neuronal function in the offspring brain.

The present study was undertaken to examine the roles of maternal polyI:C on the expression levels of proBDNF in hippocampal CA3 and CA1 regions. To investigate cognitive behavior associated with the positive symptoms of schizophrenia, we tested memory for contextual fear conditioning and an anti-proBDNF antibody was intra-infused into the CA3/CA1 of the hippocampal area. Meanwhile, LFPs were recorded at hippocampal CA3 to CA1 synapses and the phase synchronization and the index of NIF coupling between CA3 and CA1 over different frequency bands were determined. The excitatory postsynaptic currents (EPSCs) were recorded to evaluate the possible neural mechanism. Our findings provide the first direct evidence that prenatal exposure to MIA alters the expression of proBDNF and its mediated neural function, which may have profound implications for revealing the underlying neuropathology of neurodevelopmental disorders related to maternal infection.

## Experimental procedure

### Experimental animals and the maternal immune activation model of schizophrenia

Experimental procedures were performed in accordance with the Care and Use of Animals Committee of Guizhou University of Traditional Chinese Medicine (SCXK-2013-0020) and the practices outlined in the NIH Guide for the Care and Use of Laboratory Animals. Sprague-Dawley rats (Laboratory Animal Center, Academy of Military Medical Science of People’s Liberation Army) were reared in standard polypropylene ventilated cages in a colony room (12-h light-dark cycle; lights on at 7 a.m., 21 ± 2°C; 45 ± 5% humidity) with free access to tap water and standard pellet diet. Nulliparous time-mated rats (*n* = 35) were left undisturbed until treatment on the gestational day (GD) 15. Experiments were conducted during the light phase (between 1 p.m. and 5 p.m.)

Maternal treatment generally followed previously established protocols (Gibney et al., 2013; Lins et al., 2018; Lins et al., 2019). On GD15, dams were anesthetized with isoflurane (5% induction, 3% maintenance) and received a single intravenous tail vein injection of either 0.9% saline (*n* = 12) or polyI:C (4 mg/kg, high molecular weight, InVivoGen; *n* = 20). Care was taken to ensure the saline-treated dams were anesthetized for the same length of time as the polyI:C-treated dams. Body weight and rectal temperature measurements were taken again from the dams 8, 24, and 48 h after treatment. Dams were then left undisturbed until the day after the parturition. Experimenters were blind to the treatment of the animals and all experiments were conducted at about postnatal day (PND) 56 (range from 54 to 61).

### Samples and Western blotting

PolyI:C-treated offspring were anesthetized and bilaterally hippocampal CA3 and CA1 tissues were micro-dissected under a dissection microscope as previously described (An and Sun, 2018; Sun et al., 2021c; Sun et al., 2021d). As previously reported (Kosalkova et al., 2012; Brombacher et al., 2020), the tissues were homogenized in cold 0.32 M sucrose containing 1 mM HEPES solution, 0.1 mM EGTA and 0.1 mM phenylmethylsulphonyl fluoride, pH 7.4, in the presence of a complete set of protease inhibitors and a phosphatase inhibitor cocktail. The homogenized tissue was pushed through nylon cell strainers (Falcon, Corning Incorporated, NY) and centrifuged twice (3,900 rpm for 5 min and 13,200 rpm for 5 min) at 4°C and filtered through 0.45 μm filters (Millipore, Billerica, MA). The supernatant fluid was collected for determining the extracellular proBDNF concentration. Nuclear/cytoplasmic proteins were purified using a Nuclear and Cytoplasmic Protein Extraction Kit (Beyotime Biotechnology). Briefly, 100 mg of tissue was homogenized with ice-cold phosphate buffer (0.05 M; 25 mg/100 µl) containing extraction reagents A and B in 20:1 ratio (for cytoplasmic protein extraction) and phenylmethylsulfonyl fluoride (1 mM) using glass tissue homogenizers. Cytoplasmic and nuclear proteins were extracted using respective kits according to the manufacturer’s instructions. The proteins were quantified using bicinchoninic acid assay (Bio-Rad Lab) and resolved by 10%–15% SDS-PAGE. Then they were transferred onto PVDF membranes (Pall, Florida, United States) for immunoblotting. The membranes were blocked with 5% non-fat skimmed milk for 1 hour and incubated with the primary mouse anti-proBDNF (1:500; Cat#sc-65514, Santa Cruz Biotechnology) and mouse anti-β-actin (1:20,000; Cat#A5316, Sigma) overnight at 4°C. Then the membranes were incubated with horseradish-peroxidase (HRP)-conjugated secondary goat anti-mouse IgG (1:2500; Cat#31430, Thermo Fisher Scientific) incubated for 2 hours. After three washes with TBST buffer, immunoreactivity was detected by ECL Detection Kit (CWBIO, China) as our previous reports (Sun et al., 2018b; Sun et al., 2021e; Sun et al., 2022a).

### Enzyme-linked immunosorbent assay

The levels of mBDNF in the hippocampal CA1 and CA3 regions were determined using the BDNF ELISA kit (Cat#ERBDNF, Thermo Fisher Scientific) according to the manufacturer’s instructions. Briefly, the samples were incubated with pre-coated 96-well plate for 2.5 h at room temperature. Then biotin conjugate (for 60 min), Streptavidin-HRP (for 45 min), TMB substrate (for 30 min) and stop solution (for 30 min) were added and incubated at room temperature. Optical absorbance was measured using a microplate reader (Bio-Rad, United States) at 450 nm, and the concentration was calculated according to the standard curve.

### Stereotaxic surgery and microinjection

For surgery preparation, animals were anesthetized with isoflurane and fixed in a custom-made stereotaxic apparatus (SN-3, Narishige, Japan) (An et al., 2012; An et al., 2019; Sun et al., 2021f). Body temperature was maintained with a heated gel pad. Guide cannulae (22 Ga) were bilaterally implanted into the CA1 (AP: −3.3 mm, ML: ±2.2 mm, DV: 2.4–2.8 mm) or CA3 (AP: 4.2 mm, ML: 3.5 mm, DV: 2.3–2.6 mm) region. Gauge dummy cannulae (30-Ga, Plastics One Inc.), which extended 0.5 mm beyond the guide cannulae, were inserted to prevent clogging. Rats were given at least 7 days to recover from the surgery.

Infusions were performed by inserting custom needles (30 Ga) connected through PE-50 tube into an infusion pump (Harvard Apparatus), extended 1.0 mm pass the end of the cannulae. One week before the treatment, the infusion procedure was habituated on two separate days. The cleavage-resistant proBDNF (2 ng/ml; Cat#B257 Alomone Labs), TAT-Pep5 (4 ng/μl; Cat#506181, EMD Millipore), or artificial CSF (ACSF) was infused bilaterally at a rate of 0.5 μl/min/side for 2 min. The infusions were conducted 30 min prior to the detection of proBDNF expression. For fear learning, the infusions were performed 30 min before conditioning; for the memory test, the infusions were performed immediately following the conditioning. The cannulae were left for an additional 5 min to allow drug diffusion. Dose and route were chosen based on the previous studies (An et al., 2018; Sun et al., 2020; Sun et al., 2022b).

### Fear conditioning

A new cohort of 61 rats were contextual fear conditioned and tested in standard operant chambers (Coulbourn Instruments) inside sound-attenuating boxes (Med Associates) as previously described (Sun et al., 2018a; Sun et al., 2021b). To rule out the contribution of the infusions before the fear conditioning to memory consolidation, rats were separated into two subgroups (Fear learning: 24 polyI:C-treated and 6 saline-treated offspring; Memory test: 25 polyI:C-treated and 6 saline-treated offspring). For the conditioning stage, the conditioned stimulus (CS) was a tone (4 kHz, 30 s, 77 dB), which was co-terminated with a foot-shock (0.5 s, 0.5 mA) as the unconditioned stimulus (United States ). The protocol consisted of five habituation tones, followed by six tone-shock pairings. The intertrial interval varied around 2 min to prevent any association with time. The chambers were cleaned between each testing with 70% ethanol and wiped with paper towels. The memory test was conducted in the same context used for conditioning 24 h following the conditioning. Fear behavior was assessed offline from videos by measuring freezing with the exception of respiration-related movement and non-awake or rest body posture (Blanchard and Blanchard, 1969). Percentage freezing was measured during each tone presentation. Behavior was videotaped and later scored offline with a digital stopwatch by an experimenter blind to the experimental conditions as previously described (Sun et al., 2018a; Sun et al., 2019; Sun et al., 2021b) and the average percentage of freezing was calculated.

### Local field potential recordings

Independently microelectrodes were arranged in two 2 by 2 matrix using 17 μm polyimide-coated platinum-iridium (90%/10%; California Fine Wire) in a 16-gauge silica tube (World Precision Instruments). The tips of electrodes were plated with platinum to reduce impedances to 150–500 kΩ. A cannula was attached to a silica tube and its proximal open end was parallel to electrode tips. Two electrode arrays were chronically implanted: one was located at the CA1 region (AP: 3.5 mm, ML: 2.5 mm, DV: 2.0 mm) and the other one was located at the CA3 (AP: 4.2 mm, ML: 3.5 mm, DV: 2.5 mm) of the hippocampus in the ipsilateral hemisphere using previously reported procedures (An et al., 2013; An and Zhang, 2015; An and Sun, 2017a).

The recording was conducted 10 min before the behavioral test in their home-cage and during the whole behavioral test using a Digital Lynx system and Cheetah recording software (Neuralynx Inc.). LFPs were continuously sampled at 2 kHz and filtered at 0.1–1,000 Hz from each electrode. The animals’ behavior was monitored by a digital ceiling camera (Neuralynx Inc.) and sand the CCD camera’s signal was fed to a frame grabber (sampling rate, 1 MHz) with the experimental time superimposed for offline analysis.

### Phase locked value

PLV is defined to analyse the strength of phase synchronization. Extracting the phase of two signals, $\phi_{a}$ and $\phi_{b}$ were obtained. PLV is defined as following,

$$PLV=\left|\frac{1}{N}\overset{N}{\underset{j=1}{\sum }}\exp\left(i\left[\phi_{a}\left(j\Delta t\right)-\phi_{b}\left(j\Delta t\right)\right]\right)\right|$$

with N stands for the length of time series, $\frac{1}{\Delta t}$ is the sampling frequency. The value of PLV is between 0 and 1, meaning that 1 indicates fully synch and 0 no syncing at all.

### General partial directed Coherence algorithm

PDC, whose definition is based on the notion of linear Granger causality, is proposed to describe the causal relationship between multivariate time series. Its core meaning is based on the decomposition of multivariate partial coherences computed from multivariate autoregressive models. 2-Variate process PDC algorithm was introduced as follows.

Considering a two dimensional process

$$X\left(t\right):=\left[x_{1}\left(t\right)\;x_{2}\left(t\right)\right]$$ (1)

Granger causality within a 2-variate process defined by X(t) is assessed by modeling them through a vector autoregressive (VAR) model of the form:

$$X\left(t\right)=\overset{p}{\underset{r=1}{\sum }}A_{r}X\left(t-r\right)+E\left(t\right)$$ (2)

with $A_{r}=\left[\begin{array}{cc} a_{11}\left(r\right) & a_{12}\left(r\right)\\ a_{21}\left(r\right) & a_{22}\left(r\right) \end{array}\right]$

Taking the Fourier Transformation of the VAR coefficients:

$$A\left(f\right)=\overset{p}{\underset{r=1}{\sum }}A_{r}\cdot \exp\left(-i2\pi fr\right)$$ (3)

Yields a frequency-domain representation of the VAR model.

Defining the matrix: $\overset{-}{A}\left(f\right)=I-\overset{p}{\underset{r=1}{\sum }}A_{r}\cdot \exp\left(-i2\pi fr\right)=\left[\overset{-}{a_{1}}\left(f\right)\;\overset{-}{a_{2}}\left(f\right)\right]$ as the difference between the identity matrix.

And then PDC from variable $x_{j}$ to $x_{i}$ is defined as:

$$\pi_{ij}\left(f\right)=\frac{{\overset{-}{a}}_{ij}\left(f\right)}{\sqrt{{\overset{-}{a}}_{j}^{H}\left(f\right){\overset{-}{a}}_{j}\left(f\right)}}$$ (4)

It has been shown that large differences in the variances of the modeled time series can lead to distortions in the resulting PDC values (Winterhalder et al., 2005; Baccala et al., 2007; Sun et al., 2021g). To avoid this, a variation of the original PDC which is called *generalized PDC* (gPDC) (Baccala et al., 2007; Sun et al., 2021h) is presented. In gPDC, the coefficients ${\overset{-}{A}}_{ij}\left(f\right)$ are normalized by the standard deviation of the E(t) model residuals:

$$\pi_{ij}^{g}\left(f\right)=\frac{\frac{1}{\sigma_{i}}{\overset{-}{a}}_{ij}\left(f\right)}{\sqrt{\frac{1}{\sigma_{1}^{2}}{\overset{-}{A}}_{1j}\left(f\right){\overset{-}{A}}_{1j}^{H}\left(f\right)+\frac{1}{\sigma_{2}^{2}}{\overset{-}{A}}_{2j}\left(f\right){\overset{-}{A}}_{2j}^{H}\left(f\right)}}$$ (5)

The denominator in (Gustafsson et al., 2018) is a normalization that bounds the gPDC coefficients to values from 0 to 1. The choice of scaling means that $\left|\pi_{ij}^{g}\left(f\right)\right|$ measures the outflow of information from signal $x_{j}$ to signal $x_{i}$ with respect to the total outflow of information from $x_{j}$ to all signals.

### Whole-cell patch clamp electrophysiology

As previously described (An and Sun, 2017b; Li et al., 2018; Sun et al., 2021e), animals were euthanized and the brains were removed to an ice-cold solution containing 200 mM Sucrose, 1.9 mM KCl, 1.2 mM NaH_2_PO_4_, 33.0 mM NaHCO_3_, 10.0 mM glucose, 4.0 mM MgCl_2_ and 0.7 mM CaCl_2_, pH 7.3 (with an osmolarity of 300–305 mOsm). On a vibratome (VT1000S, Leica, Germany), 300-μm-thick horizontal slices were prepared, since horizontal preparations can preserve clearer layering than parasagittal or coronal ones and yield a slice attached with clear fiber projections and multiple neurons (Van Hoeymissen et al., 2020). After 60 min recovery, slices were recorded in a chamber, which was placed on an upright microscope (Zeiss Axioscope II FS plus) equipped with a ×40 water immersion objective and a visualization system (Hamamatsu Photonics) consisting of an infrared CCD camera (C7500) and the controller (C2741). The slices were perfused with a continuous flow of ACSF (95% O_2_ and 5% CO_2_) that contained 120 mM NaCl, 3.5 mM KCl, 1.25 mM NaH_2_PO_4_, 26.0 mM NaHCO_3_, 10.0 mM glucose, 1.3 mM MgCl_2_ and 2.5 mM CaCl_2_, pH 7.3. When it was necessary, the ACSF which contained anti-proBDNF antibody (2 ng/ml, or 5 ng/ml) or Pep5 inhibitor (4 ng/μl) was perfused.

Whole-cell voltage-clamp recordings were performed in pyramidal neurons of the hippocampal CA1 region using pipettes with 3–7 M resistance after being filled with pipette solution containing (in mM) 120 mM potassium methanesulfonate, 10 mM NaCl, 10 mM EGTA, 1 mM CaCl_2_, 10 mM HEPES, 5 mM ATP-Mg at pH 7.3. The pipettes were pulled using a P-97 electrode puller (Sutter Instruments). Recordings were performed at -70 mV holding potential in the presence of 10 μM bicuculline at room temperature (22 ± 1°C). Data of EPSC were recorded using an EPC-10 patch-clamp amplifier (HEKA Instruments). Signals were digitized at 10 kHz and low-pass filtered at 2 kHz and collected using Pulse software (HEKA, Germany). The frequency and amplitude of individual events were examined with Clampfit software (Molecular Devices). The recording was initiated 30 min following the drug perfusion. The recording time for the final analysis was around 15 min. Only one slice including the entire hippocampus from each rat was used for each animal because of the time required to complete the treatment and the consequent uncertainty regarding any alterations in tolerance *in vitro* beyond the time.

### Data and statistical analysis

Data were expressed as Mean **±** SEM. All analyses were performed with Neuroexplorer, Matlab (MathWorks) and SPSS 17.0 software. One-Sample Student t-tests were used to compare data from blotting tests. One-way ANOVA was used to compare the data from LFP recordings and two-way ANOVA was used to compare the data from EPSC recordings. In the behavioral test, two-way repeated measures ANOVA was applied to analyze freezing levels during the conditioning and one-way ANOVA was applied to analyze freezing levels in the memory test. Significant ANOVA results were followed up using Tukey’s *post hoc* test. *p* < 0.05 level of confidence was used in the analyses. The number of animals in each group for each test can be found in figure legends and results.

## Results

Following polyI:C injection, two dams were euthanized because they failed to recover from hypothermia and one dam was not able to breed to produce viable litters. Of the original 35 dams, offspring from a total of 32 litters were included (20 polyI:C-treated dams and 12 saline-treated dams).

### Maternal polyI:C treatment increases the expression of intracellular proBDNF in hippocampus

**FIGURE 1:**
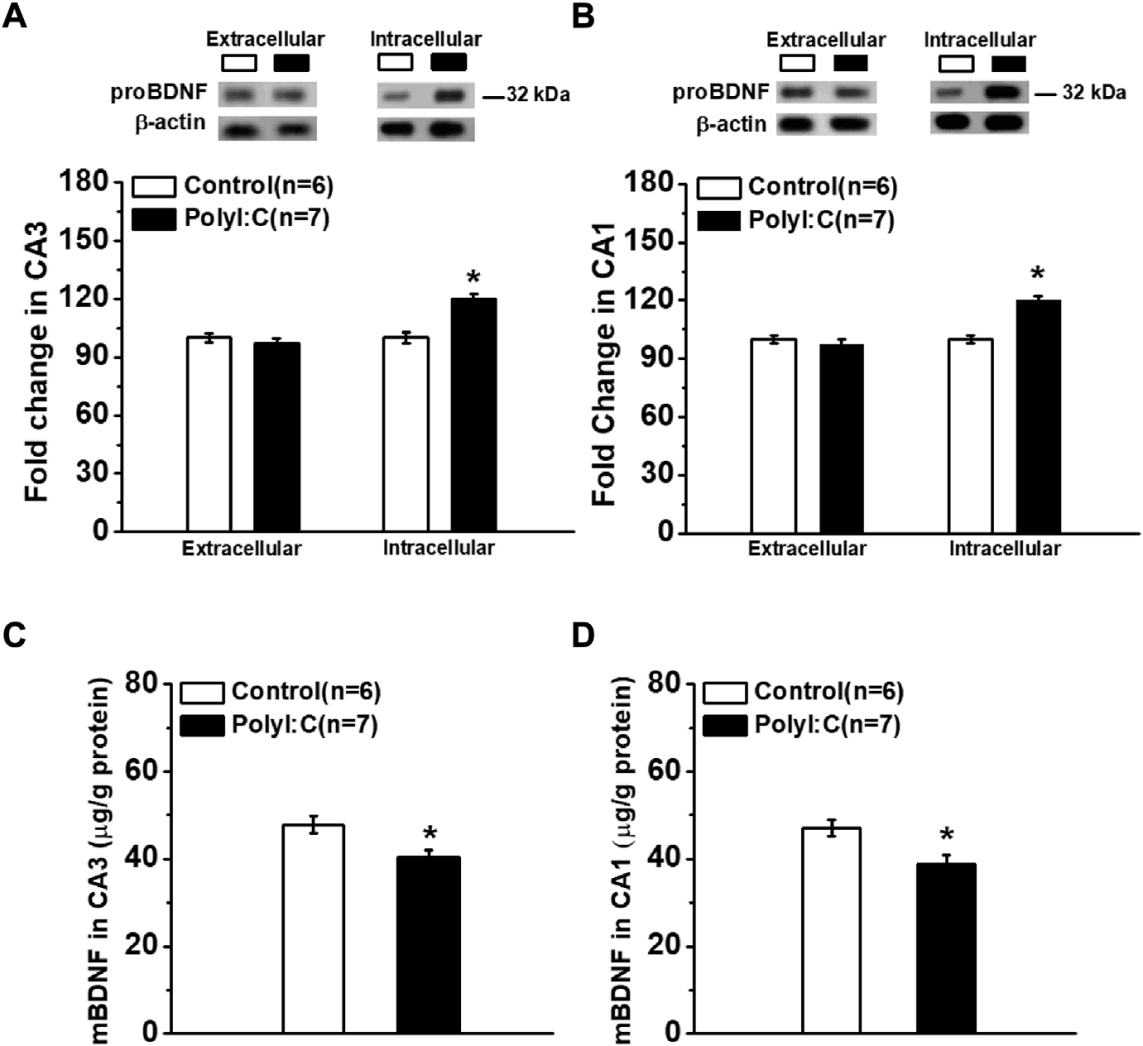
The expression of proBDNF in hippocampal CA3 and CA1 regions. Western blot analysis shows the proBDNF expression in CA3. **(A)** and CA1. **(B)** regions are abnormal increased. ELISA analysis of mBDNF concentration in CA3 **(C)** and CA1 **(D)** regions. Data are presented as mean ± SEM. *p < 0.05. Control: *n* = 6; PolyI:C: *n* = 7.

In response to polyI:C treatment, the levels of extracellular proBDNF in hippocampal CA3 (Figure 1A; *t*-test, *t*_11_ = 0.51, *p* = 0.626) and CA1 (Figure 1B; *t*-test, *t*_11_ = 0.26, *p* = 0.783) regions were comparable between polyI:C-treated and control offspring. However, the intracellular proBDNF in the CA3 area was significantly enhanced (Figure 1A; *t*-test, *t*_11_ = 2.75, *p* = 0.019). A similar pattern of expression was found in the CA1 area with statistically increased proBDNF level observed (Figure 1B; *t*-test, *t*_11_ = 2.98, *p* = 0.012). Meanwhile, the expression levels of mBDNF were declined in both CA3 (Figure 1C; *t*-test, *t*_11_ = 2.26, *p* = 0.045) and CA1 (Figure 1D; *t*-test, *t*_11_ = 2.39, *p* = 0.038) regions.

### Maternal polyI:C treatment compromises fear memory formation

**FIGURE 2:**
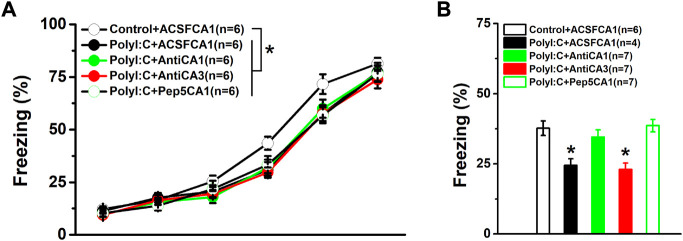
The performance in the conditioning and fear memory test. PolyI:C-treated offspring were intra -CA1 or -CA3 infused with anti-proBDNF antibody or p75^NTR^ inhibitor TAT-Pep5 30 min before the testing. The freezing levels of polyI:C-treated offspring during the training **(A)** and the memory test **(B)** are lower while blocking the activation of proBDNF/p75^NTR^ signaling can effectively reversed the impaired memory behavior. Data are presented as mean ± SEM. *p < 0.05, Control + ACSFCA1 vs. other groups in A and the group vs. Control + ACSFCA1, PolyI:C + AntiCA1 and PolyI:C + Pep5CA1 in B. Training phase: Control + ACSFCA1: *n* = 6; PolyI:C + ACSFCA1: *n* = 6; PolyI:C + AntiCA1: *n* = 6; PolyI:C + AntiCA3: *n* = 6; PolyI:C + Pep5CA1: *n* = 6. Memory test: Control + ACSFCA1: *n* = 6; PolyI:C + ACSFCA1: *n* = 4; PolyI:C + AntiCA1: *n* = 7; PolyI:C + AntiCA3: *n* = 7; PolyI:C + Pep5CA1: *n* = 7.

To identify the regional effects of the enhanced proBDNF on fear learning and memory, anti-proBDNF antibodies were injected into the CA1 or CA3 regions of PolyI:C offspring. As shown in Figure 2A, a two-way repeated measures ANOVA revealed a significant treatment effect on freezing levels during the fear acquisition (effect of treatment, *F* (4, 25) = 4.81, *p* = 0.005). The differences were found between control and ployI:C groups (Control + ACSFCA1 vs. PolyI:C + ACSFCA1, PolyI:C + AntiCA1, PolyI:C + AntiCA3 and PolyI:C + Pep5CA1, all *p* < 0.05), but no effect was found following the antibody or Pep5 infusion as the evidence by lack of difference between ployI:C groups (PolyI:C + ACSFCA1 vs. PolyI:C + AntiCA1, PolyI:C + AntiCA3 and PolyI:C + Pep5CA1, all *p* < 0.05). When the performance was compared in the memory test, the freezing level of polyI:C-treated offspring was significantly lower compared to control offspring (Figure 2B; one-way ANOVA, effect of treatment, *F* (4, 26) = 4.22, *p* = 0.009; Control + ACSFCA1 vs. PolyI:C + ACSFCA1, *p* < 0.05). Infusion anti-proBDNF antibody into CA1 (Control + ACSFCA1 vs. PolyI:C + AntiCA1, *p* < 0.05) but not CA3 (Control + ACSFCA1 vs. PolyI:C + AntiCA3, *p* < 0.05) region effectively enhanced the declined freezing level. To further determine whether p75^NTR^ served as the receptor for the proBDNF to disrupt fear memory formation, p75^NTR^ inhibitor TAT-Pep5 was infused into the CA1 region. As shown in Figure 2B, blocking the proBDNF/p75^NTR^ signaling pathway by TAT-Pep5 inhibitor could also prevent the markedly reduced freezing behavior (Control + ACSFCA1 vs. PolyI:C + Pep5CA1, *p* < 0.05).

### Maternal polyI:C treatment weakened the strength of phase synchronization and neural information flow from hippocampal CA3 to CA1 reigion

**FIGURE 3:**
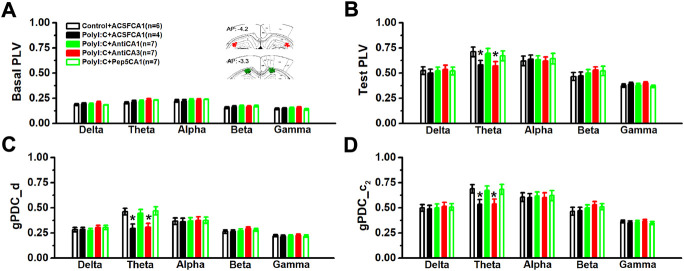
The values of PLV and the directional coupling index *d* and *c*_*2*_ between CA3 and CA1 regions. The basal value of PLV before behavioral test **(A)** and the PLV value during the contextual memory test **(B)**. The inset showing approximate locations of electrodes. Images are adapted from The rat brain atlas in stereotaxic coordinates (122, 123). Coupling direction index *d* between CA1 and CA3 **(C)** and unidirectional influence index *c*_*2*_ **(D)**. Data are presented as mean ± SEM. *p < 0.05, vs. Control + ACSFCA1, PolyI:C + AntiCA1 and PolyI:C + Pep5CA1. Control + ACSFCA1: *n* = 6; PolyI:C + ACSFCA1: *n* = 4; PolyI:C + AntiCA1: *n* = 7; PolyI:C + AntiCA3: *n* = 7; PolyI:C + Pep5CA1: *n* = 7.

To assess whether there was an abnormal baseline synchronized activity, the phase synchronization was firstly compared in home-cages 10 min before moving into the recording chambers. There was no statistical difference in the basal values of PLV (Figure 3A; two-way ANOVA, effect of treatment, *F* (4, 26) = 0.39, *p* = 0.813). Although the value of PLV in the polyI:C-treated offspring was significantly reduced in theta frequency bands compared to control group (Figure 3B; two-way ANOVA, interaction effect between treatment and band, *F* (16, 104) = 2.02, *p* = 0.018; Control + ACSFCA1 vs. PolyI:C + ACSFCA1, *p* < 0.05), intra-CA1 (PolyI:C + ACSFCA1 vs. PolyI:C + AntiCA1, *p* < 0.05) but not intra-CA3 (PolyI:C + ACSFCA1 vs. PolyI:C + AntiCA3, *p* > 0.05) infusion of anti-proBDNF antibody obviously increased the value of PLV index. Similar to abnormalities seen in phase synchronization, the directionality index *d* of NIF in CA3-CA1 pathway was decreased at theta frequency of the ployI:C-treated group (Figure 3C; two-way ANOVA, interaction effect between treatment and band, *F* (16, 104) = 2.13, *p* = 0.012; Control + ACSFCA1 vs. PolyI:C + ACSFCA1, *p* < 0.05). Furthermore, the strength of unidirectional coupling index c_2_, which reflects the unidirectional coupling from CA3 to CA1 region, was significantly diminished at theta frequency compared polyI:C + ACSFCA1 to Control + ACSFCA1 offspring (Figure 3D; two-way ANOVA, interaction effect between treatment and band, *F* (16, 104) = 2.09, *p* = 0.14; Control + ACSFCA1 vs. PolyI:C + ACSFCA1, *p* < 0.05). Both microinfusion of anti-proBDNF antibody and blocking proBDNF/p75^NTR^ pathway in area CA1 could effectively enhance the strength of NIF between CA3 and CA1 regions (PolyI:C + ACSFCA1 vs. PolyI:C + AntiCA1 and PolyI:C + Pep5CA1, both *p* < 0.05) and the unidirectional coupling (PolyI:C + ACSFCA1 vs. PolyI:C + AntiCA1 and PolyI:C + Pep5CA1, both *p* < 0.05). However, the restorative effects were not found when the treatments were performed in subfield CA3 (PolyI:C + ACSFCA1 vs. PolyI:C + AntiCA3, both *p* > 0.05).

### Ampllitude but not frequency of sEPSCs was depressed in CA1 pyramidal neurons of polyI:C-treated offspring

**FIGURE 4:**
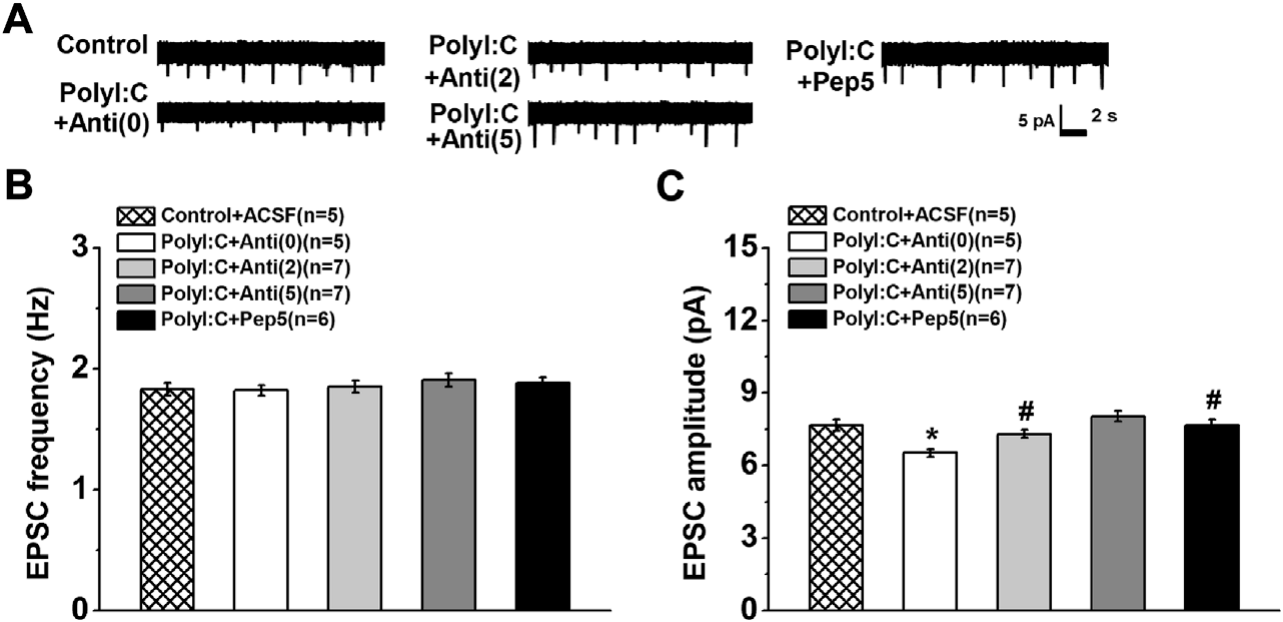
**(A)** The changes in EPSC of prymidal CA1 neurons. Typical consecutive sample traces of sEPSCs from each group (Top). The frequency of sEPSC **(B)** is not altered but the amplitude of sEPSC **(C)** is decreased in polyI:C-treated offspring. Incubation with anti-proBDNF antibody or TAT-Pep5 inhibitor can significantly enhance the declined amplitude. Data are presented as mean ± SEM. *p < 0.05, vs. Control + ACSFCA1, PolyI:C + AntiCA1 and PolyI:C + Pep5CA1. Control + ACSFCA1: *n* = 5; PolyI:C + ACSFCA1: *n* = 5; PolyI:C + AntiCA1: *n* = 7; PolyI:C + AntiCA3: *n* = 7; PolyI:C + Pep5CA1: *n* = 6.

The mean frequency of sEPSCs was not influenced by maternal polyI:C treatment (Figures 4A,B one-way ANOVA, effect of treatment, *F* (4, 25) = 0.96, *p* = 0.447). Although an enhanced effect on CA1 neurons of polyI:C-treated offspring was found when anti-proBDNF antibody was incubated at the concentration of 5 ng/ml, it failed to reach significance. However, the amplitude was significantly suppressed in maternal polyI:C treatment group (Figure 4C one-way ANOVA, effect of treatment, *F* (4, 25) = 3.82, *p* = 0.015; Control + ACSF vs. PolyI:C + Anti (0), *p* < 0.05). Moreover, anti-proBDNF antibody obviously reversed the depressive effects in a dose-response manner (PolyI:C + Anti (0) vs. PolyI:C + Anti (2) and PolyI:C + Anti (5), both *p* < 0.05; PolyI:C + Anti (2) vs. PolyI:C + Anti (5), *p* < 0.05). Meanwhile, TAT-Pep5 inhibitor could also enhance the reduction in the amplitude of polyI:C-treated offspring (PolyI:C + Anti (0) vs. PolyI:C + Pep5, *p* < 0.05).

## Discussions

In this study, we found that MIA by prenatal polyI:C treatment abnormal enhanced the expression of proBDNF in both hippocampal CA1 and CA3 regions of adult offspring. Behavioral results found the polyI:C-treated offspring displayed learning and contextual memory deficits. Intra-hippocampal infusions of anti-proBDNF antibody and p75^NTR^ inhibitor into the CA1 area could obviously mitigate phase synchronization and the directional index of NIF of polyI:C-treated offspring leading to the improvement of memory formation. Furthermore, the elevation of proBDNF induced a reduction of the amplitude of EPSCs in the CA1 pyramidal neurons, implying the alternations in postsynaptic information processing. Our findings identify the first time proBDNF signaling involved in prenatal polyI:C-induced cognitive impairments and neural coupling dysfunction.

Although alterations in the expression of mBDNF in the brain of patients with schizophrenia have been reported and thought to play an important role in the pathophysiology of major psychiatric disorders, the expression levels of mBDNF, which are generated from the resulting enzymatic cleavage and conversion of proBDNF, was varied reported. For example, increased levels of mBDNF protein in the anterior cingulate cortex and hippocampus of patients with schizophrenia were previously found (Takahashi et al., 2000; Durany et al., 2001). However, others reported a significant reduction in BDNF mRNA and mBDNF levels in the hippocampus of suicide victims (Weickert et al., 2003; Karege et al., 2005). Recently, mBDNF in the placenta was insignificant and remained unaffected following the activated maternal immune system (Arutjunyan et al., 2020), suggesting that further studies are needed to better understand the potential involvement of BDNF and its signaling. Our results showed that polyI:C-induced MIA resulted in an abnormal enhancement in the expression of proBDNF in the hippocampal regions, including CA3 and CA1. Similarly, the activation of its receptor p75^NTR^ level was found to be elevated on PND 20 (Lin and Wang, 2014). Recently, we found a peak expression of proBDNF in the hippocampus during the postnatal period and a steady lower level in adulthood rats (Sun et al., 2021a). Anatomical and histological observation also found that the up-regulation of proBDNF/mBDNF ratio induced synaptic plasticity impairment, and cognitive decline in depressive episodes and aged (Diniz et al., 2018; Wang et al., 2021), indicating BDNF has bidirectional effects on neuronal function depending on the cleavage of proBDNF (Barnes and Thomas, 2008; Menshanov et al., 2015; Wang et al., 2021).

Our behavioural findings showed that MIA, following polyI:C administration, impaired contextual fear memory consolidation processes. It was consistent with previously published research utilizing bacterial endotoxin lipopolysaccharide and the viral mimetic polyI:C (Huang et al., 2010; Kranjac et al., 2012a; Kranjac et al., 2012b; Bao et al., 2022). Furthermore, treatment of p75^NTR^ inhibitor TAT-Pep5 and anti-proBDNF antibody in the CA1 but not CA3 area could prevent memory deficits of MIA adult offspring. Our study confirms that the activation of proBDNF-p75^NTR^ pathway in the hippocampus is involved in schizophrenia-like behavior abnormalities in offspring after MIA. Thus, proBDNF-p75^NTR^ signaling would represent a potential therapeutic and preventive target for schizophrenia. Somewhat of a surprise to us, we did not observe significant reversal effects of anti-proBDNF antibody on fear learning after MIA exposure. An explanation of our findings would be that activation of proBDNF/p75^NTR^ signaling improves correct responses and reduce error rates during the early phase of reversal training, with no effect on memory acquisition (Sun et al., 2020). Indeed, this conclusion is consistent with our prior data that infusions of anti-proBDNF antibodies did not affect animals’ performance during the training stage but had trouble in the adaption and acquisition of learning flexible behavior (An et al., 2018). Moreover, the antibody and p75^NTR^ inhibitor had no effect on memory retrieval when they were administered beyond the time window for memory consolidation and reconsolidation (Leutgeb et al., 2004; Huang et al., 2010), rendering the impossibility that the improved memory attributed to their effects on memory retrieval. Therefore, our results indicate that the deleterious impacts of prenatal polyI:C treatment on memory consolidation of contextual fear memory are mediated by abnormally increased proBDNF in the hippocampus.

Hippocampal place cells, located in the CA1 region, are the neurons with spatially localized activities (Henriksen et al., 2010; Koh et al., 2016). We found that the abnormal elevation of the proBDNF level in the CA1 but not the CA3 region contribute to memory deficits. Consistently, the reduction in inhibitory effects specifically in the CA1 of the hippocampus could impact trace fear memory (Brigman et al., 2010). Molecular signaling in the CA1 has also been found to be important for trace fear memory (Huang and Morozov, 2011; Soltesz and Losonczy, 2018). Interestingly, although pyramidal neurons in the hippocampal CA3 express high levels of BDNF, this BDNF does not affect hippocampal oscillations but the oscillation power can be facilitated in the CA1 area by attenuating the expression of 5-Hydroxytryptamine type 3 receptors (Schobel et al., 2009). Our results coincided that hippocampal CA1, but not other regions, is differentially targeted by schizophrenia and related psychotic disorders (Knuesel et al., 2014). Thus, it is interesting to postulate that CA1 hypermetabolism may be driving dysfunction in other brain regions in the established illness.

The inflammatory response in the developing hippocampus following maternal infection may influence the formation and development of neural circuits (Maynard et al., 2001; Je et al., 2013). Actually, proBDNF and its high-affinity receptor p75^NTR^ signaling negatively regulate neurodevelopment, regulating neuronal apoptosis and remodeling, including axonal retraction and synaptic elimination (Yang et al., 2009; Yang et al., 2014; Hill et al., 2016). Therefore, the increased proBDNF alters the connectivity of functional circuits underlying the cognitive deficits related to schizophrenia and other neurodevelopmental disorders. Specifically, BDNF is thought to influence neuronal network activities, such as theta oscillations (Ducharme et al., 2012), that are critical for ongoing information processing and modification of synaptic efficacy (Mascetti et al., 2013; Park and Poo, 2013). For example, Bdnf-e4 mice with an exaggerated enhancement in hippocampal low theta power were shown to impair freezing behavior (Ducharme et al., 2012). It was in agreement with the observation of hippocampal hyperactivation in individuals with the BDNF rs6265 (Val66Met) polymorphism (Nagappan et al., 2009). Our previous studies suggest that proBDNF-mediated low-frequency oscillations play a prominent role in preventing fear memory processing, with special emphasis on the facilitation of memory extinction (Sun et al., 2018a; Sun et al., 2019). It is worth noting that theta burst stimulation, which can mimic continuous theta-burst firing during learning, is preferred to induce predominant cleavage of proBDNF (Edelmann et al., 2015). Furthermore, theta bursts of postsynaptic action potentials (APs) preceding presynaptic stimulation trigger BDNF-dependent postsynaptic timing-dependent potentiation (Lynch, 2004), which is essential for memory formation (Crabtree and Gogos, 2014). Synaptic plasticity alters the strength of information flow between presynaptic and postsynaptic neurons, thereby regulating the likelihood that APs in presynaptic neurons lead to APs in postsynaptic neurons (Kallupi et al., 2014). Consistently, our results showed that the amplitude of EPSCs in the CA1 region declined in MIA offspring, suggesting the disruption of postsynaptic information processing (An and Sun, 2017b; Speers and Bilkey, 2021). It has been demonstrated that NMDAR-dependent intrinsic excitability in CA1 pyramidal neurons from MIA offspring was markedly reduced, which caused the detrimental effect on spatial memory impairments (Escobar et al., 2011; Zeraati et al., 2021). Further investigation demonstrated the possible mechanism by which environmental enrichment relieves the symptoms of cognitive deficits. Environmental enrichment dampens the activity of the HPA axis and enriched at postsynaptic sites of excitatory synapses, including postsynaptic density protein 95 (PSD95) and NMDAR-GluN2B (Zhao et al., 2020; Ma et al., 2022). Moreover, LTD was unchanged by acute block of mBDNF signaling while GluN2B-mediated synaptic depression was suppressed by blocking proBDNF/p75^NTR^ signaling (Ramser et al., 2013; Sun et al., 2021a). Meanwhile, local inhibition of p75^NTR^ signaling in the amygdala during or after fear extinction training resulted in memory extinction impairments (Ramser et al., 2013), suggesting that proBDNF/p75^NTR^-mediated neuronal excitability plays a pivotal role in memory decay. Additionally, the hypofunction of calcineurin in schizophrenia animals, which displayed dysfunction in BDNF trafficking (Pineda et al., 2009; Suh et al., 2013), had the potential to alter synaptic plasticity and memory behavior and result in profound disruptions of information processing (Miyakawa et al., 2003; Cottrell et al., 2013; Tamura et al., 2018). Although we found that polyI:C offspring displayed a reduction in the direction of information flow at the theta oscillations, gamma oscillations, which have previously been found to be involved in spatial memory processes (Nyhus and Curran, 2010; McNally and McCarley, 2016), were not markedly changed in the CA3-CA1 pathway. Indeed, gamma oscillations are shown to nest within the overlying theta oscillations during memory tasks (Lega et al., 2016; Kight and McCarthy, 2017). Possibly, the disparities found here represent disruptions of this cross-frequency coupling. The mechanisms underlying this effect needed to be further determined.

In summary, our findings clearly demonstrate that the abnormal enhancement in hippocampal proBDNF secretion disrupts proBDNF/p75^NTR^ signaling mediated neural information processing leading to impairing long-term contextual memories in offspring following maternal polyI:C exposure. Further studies are required to testify if changes in proBDNF expression occur in other brain areas relevant to spatial cognition. Moreover, given the characteristic relationship between neurotrophins and estrogen (Paxinos and Watson, 1997; Paxinos and Watson, 1998; Srivastava et al., 2013), additional experiments are needed to elucidate whether similar alterations observe in females.

## Data Availability

The datasets presented in this study can be found in online repositories. The names of the repository/repositories and accession number(s) can be found in the article/supplementary material.

## Glossary

ACSF: artificial cerebrospinal fluid
AP: action potential
BBB: blood-brain barrier
CA1: Cornus Ammonis 1
CA3: Cornus Ammonis 3
CCD: charge-coupled device
CNS: Central nervous system
CS: conditioned stimulus
DG: dentate gyrus
dsRNA: double-stranded RNA
EEG: electroencephalogram
EPSC: Excitatory postsynaptic current
NMDAR-GluN2B: glutamate receptor NMDA-type subunit 2B
gPDC: general partial directed coherence
HRP: horseradish-peroxidase
IL-1β: Interleukin-1β
IL-1R1: IL-1β receptor type 1
LFP: local field potential
LTD: long-term depression
LTP: long-term potentiation
mBDNF: mature brain-derived neurotrophic factor
MIA: maternal immune activation
NIF: neural information flow
NMDAR: N-methyl-D-aspartate receptor
p75^NTR^: p75 neurotrophin receptor
PDC: partial directed coherence
PLV: phase locked value
PND: postnatal day
PolyI:C: Polyinosinic:polycytidylic acid
proBDNF: precursor of brain-derived neurotrophic factor
PSD95: postsynaptic density protein 95
PVDF: polyvinylidene difluoride
PV immunoreactivity: parvalbumin immunoreactivity
TBST: Tris buffered saline Tween
TrkB: tyrosine kinase receptor B
US: unconditioned stimulus
VAR: vector autoregressive
